# Effect of a Short Messaging Service (SMS) intervention delivered to caregivers on energy, nutrients, and food groups intake in infant participants of the WIC program

**DOI:** 10.3389/fpubh.2022.986330

**Published:** 2022-09-29

**Authors:** Alison K. Macchi, Jinan Banna, Stephanie Moreira, Maribel Campos, Cristina Palacios

**Affiliations:** ^1^Department of Dietetics and Nutrition, Robert Stempel College of Public Health and Social Work, Florida International University, Miami, FL, United States; ^2^Department of Human Nutrition, Food and Animal Sciences, College of Tropical Agriculture and Human Resources, University of Hawaii at Manoa, Honolulu, HI, United States; ^3^Center for Community Outreach for Health Across the Lifespan (COHeAL), University of Puerto Rico, San Juan, PR, United States

**Keywords:** Short Message Service (SMS), intervention, infant, energy consumption, nutrient intake, food groups and beverages consumption

## Abstract

**Objectives:**

To test the effect of a weekly test message (SMS) intervention for improving feeding practices on infant intake of energy, nutrients, and specific food groups.

**Methods:**

This study was a multi-site, randomized clinical trial, in 202 caregivers of healthy term infants participating in either the Puerto Rico or Hawaii WIC program. Participants were randomized to receive weekly SMS about either infant's general health issues (control) or SMS for improving feeding practices (intervention) to complement WIC messages for 4 months. Anthropometrics and demographics were assessed at baseline. A validated infant food frequency questionnaire was assessed at the four-month visit to assess intake of general food groups. Data was summarized as median (25th and 75th percentiles) or percentage and differences between study arms was compared using Mann Whitney or chi-square.

**Results:**

A total of 163 participants completed the study (*n* = 84 control and *n* = 79 intervention). Baseline characteristics were similar between both groups. At 4–6 months of age, compared to the control group, the intervention group had a significantly higher intake of total grains (0.28 oz; 0.00, 0.60; *P* = 0.033), protein (13.5 g, 10.5, 18.3; *P* = 0.022), calcium (472 mg; 418, 667; *P* = 0.012), and zinc (4.39 mg; 2.61, 6.51; *P* = 0.028). No differences were seen in the other food groups, including breastmilk.

**Conclusions for practice:**

Feeding SMS to complement WIC messages led to higher intakes of some key nutrients but did not have an overall improvement in the intake of food groups. Messaging also did not delay the introduction of complimentary foods or improve breastfeeding rates. Future studies should evaluate the use of more intensive SMS interventions for continued care between WIC visits.

## Introduction

The first 2 years, or 1,000 days (conception-24 months), of an infant's life are crucial for healthy growth and development (1). Infant obesity and rapid weight gain increases the risk of obesity later in life (2–4). Current evidence suggests a higher risk if the rapid weight gain occurs in the first 6 months (5). Compared to Whites and African Americans, infant obesity is higher among Hispanics (14.8%) and Native Hawaiians (11.4%) (6). Additionally, Puerto Rico (PR) and Hawaii (HI) lead the U.S. in several chronic diseases, such as diabetes and hypertension (7). Unhealthful lifestyles and poor health education, in addition to other social determinants of health, may underlie many of these chronic diseases (i.e., obesity, diabetes, hypertension) as evidenced by the low intake of fruits, vegetables, low-fat dairy products and fiber in these populations (8–11) and the low diet quality, even among infants (12).

Many factors, such as socioeconomic, biological, and lifestyle (parental feeding practices and diet quality), influence infant weight gain and infant obesity (5, 13). Parental feeding practices, such as early introduction of complimentary food before 6 months of age is associated with an increased risk of obesity in infants (14). Infant diet quality is also largely affected by the introduction of discretionary foods, defined as energy dense, low nutrient food items, which include juices and salty snacks (15, 16). Discretionary food use in infants also increases the risk of obesity. In addition, introducing discretionary foods increase unnecessary caloric intake and take away from necessary nutrients found in breastmilk and formula (15). Both practices of early introduction of complementary foods discretionary foods are commonly seen in Hispanic and disadvantaged populations (17).

As discussed, some parental feeding practices may influence infant energy and nutrient intakes and food groups intake in infants. Energy and nutrient recommended intakes have been established in the US for the population, including infants, by the Institute of Medicine as Dietary Reference Intakes (DRI) (18). Some of these key nutrients are carbohydrates (60 g/d), fat (31 g/d), protein (9.1 g/d), vitamin D (10 μg/d), all B vitamins, calcium (200 mg/d), iron (0.27 mg/d), and zinc (2 mg/d). These levels ensure that infants grow adequately and avoid nutrition related diseases. However, only certain dietary recommendations have been established by the 2020–2025 Dietary Guidelines of America (DGA) for infants to meet these DRIs (19). For those under 6 months of age, recommendations are basically focused on breastmilk or formula. However, for infants 6–12 months of age, dietary recommendations are still in development (20), as this is a complex task. In Puerto Rico, recommendations were recently published for infants in collaboration between the Department of Health in Puerto Rico and the Pan American Health Organization (21). Starting at 12 months of age, where solid foods are a major part of an infant's diet, the DGA recommendations have been recently established ranging from 1 to 3 oz per food group per day.

To our knowledge, there is only one study to date analyzing if infant dietary patterns are adequately meeting the DRI in the U.S. Using data from the National Health and Nutrition Examination Survey (NHANES) from 2009 to 2012, a study showed that most infants 0-12 months in the US met the DRIs, although there were some nutrients that were consumed below these levels, such as vitamin D (22). Also, there are limited studies evaluating educational intervention to improve foods and nutrients intake in infants 0–6 months, particularly those using technology and among low-income families. Educational interventions *via* short message system (SMS) have been shown to be effective at improving overall health outcomes among low-income new mothers (23). Low-income mothers are at a higher risk for having overall poorer infant health outcomes and higher hospitalization rates in infants during the first months of life (24). Despite bearing a higher degree of burden to sustain continuity of care through traditional methods, current trends support use of SMS as a reliable method of engagement with disadvantaged mothers. SMS-based interventions can also enhance education received from government funded programs, such as WIC programming, reinforcing education between appointments.

The purpose of this study was to determine the effect of a weekly SMS intervention for improving feeding practices aimed at caregivers of infants participating in WIC programs in Puerto Rico and Hawaii on infant intake of energy, nutrients, and specific food groups.

## Materials and methods

### Design

This study was a 4-month, multi-site, randomized control clinical trial involving weekly SMS sent to infant caregivers participating in the WIC program in Puerto Rico (PR) and Hawaii (HI). Weekly SMS focused on improving infant feeding practices (intervention) or on general infant health issues (control). Details of the study methods have been previously published (25).

### Participant recruitment and randomization

A total of 202 caregivers of infants 0–2 months old who were participating in the WIC program in PR (*n* = 100) and HI (*n* = 102) were recruited in 2017. Participants were recruited from two WIC clinics in PR and 4 WIC clinics in HI. WIC clinics were selected due to their availability and accessibility to the investigators. Eligible participants included caregivers who were 18 years old or older, responsible for infant care, owners of a cellular phone capable of receiving unrestricted SMS and were willing to participate for the whole duration of the study. Participants were excluded if the infant had a special diet, limited mobility, were born pre-term (< 37 weeks), were small or large for their gestational age (birthweight < 10th or > 90th percentile), unable to consent to participate, unwilling to be randomized, or were not able to read. The institutional review boards at University of Puerto Rico and University of Hawaii approved study procedures and participants signed an informed consent form.

Once consented, an equal number of caregivers were randomized into the control arm (SMS about general infant health issues) or intervention arm (SMS for improving feeding practices) of the study. Randomization was conducted using random block sizes (2, 4, or 6) with 26 total blocks. As they were recruited, participants were allocated an ID sequentially. This ID was matched with a computer-generated list of randomization numbers and corresponding IDs. Those who were randomized into the intervention group were additionally assigned to either a lactation or formula intervention group, based on their initial feeding status. Participants received $50 for completing the study.

### Intervention

Details of the intervention have been previously published (25). Briefly, intervention SMS were sent once a week for 4 months automatically using a web-based SMS messaging platform. Messages were approximately 35–50 words long and written in a 5th grade level literacy in Spanish (PR) and English (HI) and focused on reinforcing WIC messages on breastfeeding, preventing overfeeding, delaying introduction of solid foods, and delaying and reducing baby juice consumption. The Trans Theoretical Model (TTM) of health behavior change was used to create these messages (26). The control group received messages focused on general infant health issues with a similar frequency. Both groups continued to receive the WIC standard of care (27).

### Measures

#### Baseline demographics

A sociodemographic and general health questionnaire was administered at baseline. Caregivers reported information on age, gender, education, occupation, gestational age, use and availability of a breast pump, breast feeding support, infant's age and sex, type of pediatric center, WIC center, and immunization records.

#### Food frequency questionnaire

A validated food frequency questionnaire (FFQ) was administered at the first visit (0–2 months of age) and second visit (4–6 months of age) (28). The FFQ, which is specifically validated for infants and toddlers, includes 52 food items. For each item, parents were asked to specify the frequency that their infant consumed each food in the last 7 days and the amount. The frequency of consumption of each food item was multiplied by the serving size to obtain the total food consumed per day and it was reported in ounces per day. Then, the foods were grouped in the following categories: all milks (which include formula, breastmilk, and other milks), total grains, whole grains, refined grains, protein rich foods, vegetables, fruits, juices, and salty snacks. To calculate breastmilk intake, data from the Feeding Infants and Toddlers Study was used (29). FITS is the current largest cross-sectional survey of caregivers of children from 0–48 months of age in the US. Based on data from FITS, the average total daily milk intake volume was set at 26.4 for infants aged 0–6 months. If infants were partially breastfed, the ounces in formula were subtracted from the total daily milk intake volume and what remained was categorized as breastmilk intake. If the daily volume of formula exceeded the estimated total daily milk intake volume, a rule of three ounces per feeding was implemented, which was also derived from FITS (29). For energy and nutrients calculations, we used the previously created food database as previously described and published. In brief, energy and nutrients content of each food item per one ounce obtained using the Nutrition Data System for Research (30) was multiplied by the total amount consumed by the infant per day. Then, the energy and nutrients amounts were summed for all food items to obtain total values per day.

### Statistical plan

Baseline characteristics were summarized as median (25th and 75th percentiles) for continuous variables and percentage for categorical variables and compared between the two study arms using Mann Whitney U tests for continuous variables and chi-square test for categorical variables. Energy, nutrients, and food groups intake were compared between groups using Mann Whitney U-tests, controlling for site (HI vs. PR) and number of children. SPSS v 20 was used for all statistical analysis. A *P*-value of < 0.05 was used to determine significance.

## Results

A total of 202 eligible participants were randomized into the study groups (100 in control group and 102 in intervention group) and 84 and 79, respectively, completed all food frequency questionnaires. The reasons for not completing were that participants did not respond to our messages or calls to complete the final visit. There were no significant differences in baseline characteristics between groups (Table 1). This pattern persisted irrespective of site, implying that the randomized allocation to the groups was properly performed in both sites. Overall, the median caregiver age was 28 (23.0, 31.0) years in the control and 26 (22.5, 30.0) years in the intervention group. Participants were majority Hispanic ethnicity (52%) and had less than college education (45%). Most participants had more than one child (62%), although information on whether they had previously participated in WIC programming was not obtained. The median maternal pre-pregnancy BMI was in the healthy range. For both groups, about half of the infants were female (48%), had a median age of 5 (4.0, 5.0) months at follow up visit. Median BMI for age z-score for both groups were in the healthy range.

**Table 1:** Socio-demographics and health characteristics of the sample.

**Characteristics**	**Control (*n =* 84)**		**Intervention (*n =* 79)**	
	**Median**	**25th, 75th percentile**	**Median**	**25th, 75th percentile**
***Caregiver***				
Age (years)	28.0	23.0, 31.8	26	23.0, 30.0
Ethnicity and race, *n* (%)				
Asian		11 (13.1%)		13 (16.5%)
Mixed/unknown		5 (6.0%)		5 (6.3%)
Black		3 (3.6%)		3 (3.8%)
Hispanic		49 (58.3%)		44 (55.7%)
Native Hawaiian		2 (2.4%)		2 (2.5%)
Pacific Islander		2 (2.4%)		4 (5.1%)
White		12 (14.3%)		8 (10.1%)
Education		31 (36.9%)		
Less than college, *n*, %				37 (46.9%)
Some college, *n*, %		21 (25.0%)		19 (24.1%)
College or higher, *n*, %		32 (38.1%)		20 (29.1%)
Parity			
Primiparous, *n*, %		32 (38.1%)		29 (36.7%)
2 or more, *n*, %		52 (61.9%)		50 (63.3%)
Pre-pregnancy BMI (kg/m2)	24.4	21.6, 29.7	25.3	21.5, 30.7
***Infant***				
Gender (female), *n*, %		43 (51.2%)		41 (51.9%)
Age (at follow-up, months)	5.0	4.0, 5.0	5.0	4.0, 5.0
Weight for age z score	0.0	−0.63, 0.62	0.13	−0.85, 0.53
Length for age z score	0.48	−0.48, 1.83	0.48	−0.48, 1.83
BMI for age z score	−0.41	−1.60, 0.30	−0.31	−1.29, 0.53

Table 2 shows baseline and follow-up median macro- and micro-nutrients intake in both study arms. Due to the nature of dietary changes in infants from 0 to 6 months, baseline and 4–6 months follow-up intake could not be compared, so intake was assessed separately at each timepoint between groups. Study retention at the follow-up visit was 85% (*n* = 84 in the control group and *n* = 79 in the intervention group). No significant differences in macro and micro-nutrients intake were found between both groups at baseline. At 4–6 months, the intervention group had a significantly higher intakes of protein, calcium, and zinc. However, at both baseline and 4–6 months, infants met the DRIs for all these nutrients.

**Table 2:** Energy and nutrients intake at baseline (0–2 months) and at the follow-up visit (4–6 months).

**Energy and nutrients**	**Birth-2 months**			**4–6 months**		
	**Control (*n =* 84)**	**Intervention (*n =* 79)**		**Control (*n =* 84)**	**Intervention (*n =* 79)**	
	***Median (25th,75th)***	***Median (25th,75th)***	**P-value**	***Median (25th,75th)***	***Median (25th,75th)***	***p*-value**
**Calories (kcal)**	581 (521, 604)	578 (528, 601)	0.654	579 (546, 641)	603 (568, 795)	0.080
**Protein (g)**	12.5 (11.6, 13.2)	12.9 (11.9, 13.5)	0.363	11.05 (10.2, 15.6)	13.5 (10.5, 18.3)	0.022*
**Carbohydrates (g)**	57.4 (53.7, 59.9)	57.5 (53.9, 59.5)	0.902	57.31 (56.2, 72.6)	62.6 (56.2, 97.4)	0.119
**Fat (g)**	42.5 (30.1, 50.2)	41.7 (34.1, 50.4)	0.776	35.88 (30.8, 36.6)	36.9 (31.9, 39.3)	0.292
**Fiber (g)**	0.07 (0.00, 0.19)	0.08 (0.00, 0.20)	0.520	0.21 (0.00, 0.48)	0.30 (0.00, 0.86)	0.079
**Sugar (g)**	48.5 (38.7, 50.3)	44.2 (38.0, 50.3)	0.298	55.72 (42.6, 57.3)	56.16 (44.4, 62.9)	0.065
**Vitamin D (mcg)**	2.71 (0.70, 6.16)	3.05 (0.70, 6.03)	0.619	3.89 (0.80, 9.57)	6.67 (0.85, 11.3)	0.102
**Calcium (mg)**	377 (353, 421)	386 (360, 439)	0.349	429 (398, 571)	472 (418, 667)	0.012*
**Iron (mg)**	2.46 (0.00, 6.67)	2.81 (0.00, 6.62)	0.553	4.21 (0.00, 10.6)	8.42 (0.00, 12.6)	0.090
**Zinc (mg)**	2.64 (2.22, 3.72)	2.85 (2.22, 3.99)	0.490	3.09 (2.51, 5.4)	4.39 (2.61, 6.51)	0.028*

Mann Whitney U-test; *, P < 0.05.

Table 3 shows baseline and follow-up median food groups intake in both study arms. At both time points, infants were only consuming milks and grains. At baseline, no significant differences in the intakes of milk and grains were found between both groups. At 4–6 months, milk intake was similar between groups, but intake of grains was significantly higher in the intervention group (0.28; 0.00, 0.60 ounces) compared to the control group (0.16; 0.00, 0.36 ounces; *p* < 0.05). When the individual foods were reviewed from the infant FFQ, we noticed that the major contributors to total grains were cereals added to milk in the infant bottle.

**Table 3:** Food groups intake at baseline (0–2 months) and at the follow-up visit (4–6 months).

	**Birth-2 months**			**4–6 months**		
	**Control (*n =* 84)**	**Intervention (*n =* 79)**		**Control (*n =* 84)**	**Intervention (*n =* 79)**	
	***Median (25th,75th)***	***Median (25th,75th)***	***p*-value**	***Median (25th,75th)***	***Median (25th,75th)***	***p*-value**
**All milks (oz)**	26.4 (26.4 26.4)	26.4 (26.4, 26.4)	0.914	26.4 (26.4, 28.0)	26.4 (26.4, 35.0)	0.082
*Formula (oz)*	7.00 (0.00, 19.0)	8.00 (0.00, 18.0)	0.558	9.00 (0.00, 28.0)	16.0 (0.00, 30.5)	0.138
*Breastmilk (oz)*	20.3 (0.59, 26.4)	18.4 (6.00, 26.4)	0.665	16.5 (0.00, 26.4)	2.69 (0.00, 26.4)	0.272
**Total grains (oz)**	0.07 (0.00, 0.19)	0.08 (0.00, 0.20)	0.520	0.16 (0.00, 0.36)	0.28 (0.00, 0.60)	0.033*
*Whole grains (oz)*	0.00 (0.00, 0.00)	0.00 (0.00, 0.00)	1.00	0.00 (0.00, 0.00)	0.00 (0.00, 0.00)	0.636
*Refined grains (oz)*	0.07 (0.00, 0.19)	0.08 (0.00, 0.18)	0.560	0.16 (0.00, 0.34)	0.24 (0.00, 0.42)	0.128
**Protein rich foods (oz)**	0.00 (0.00, 0.00)	0.00 (0.00, 0.00)	1.00	0.00 (0.00, 0.00)	0.00 (0.00, 0.00)	0.302
**Vegetables (oz)**	0.00 (0.00, 0.00)	0.00 (0.00, 0.00)	1.00	0.00 (0.00, 0.00)	0.00 (0.00, 0.00)	0.525
**Fruit (oz)**	0.00 (0.00, 0.00)	0.00 (0.00, 0.00)	1.00	0.00 (0.00, 0.00)	0.00 (0.00, 0.00)	0.207
**Juices (oz)**	0.00 (0.00, 0.00)	0.00 (0.00, 0.00)	1.00	0.00 (0.00, 0.00)	0.00 (0.00, 0.00)	0.728
**Salty snacks (oz)**	0.00 (0.00, 0.00)	0.00 (0.00, 0.00)	1.00	0.00 (0.00, 0.00)	0.00 (0.00, 0.00)	0.302

Mann Whitney U-test; *, P < 0.05.

## Discussion

The study intervention, which used weekly SMS to improve feeding practices in infant participants of WIC, resulted in significantly higher intakes of proteins, calcium, zinc, and total grains in infants at the follow-up visit compared to the control group. There were no significant differences in energy, other nutrients, and other food groups. Total grains came mainly from cereals added to the milk bottle.

Even though the intervention resulted in significantly higher intakes of key nutrients (proteins, calcium, and zinc), all infants met the current DRIs for key nutrients. Since the recommendations for specific food group intakes for infants in the first year of life are still in development, the results from this study provides some insights about how their dietary patterns are meeting the DRIs for this group.

While the SMS intervention was successful in increasing some nutrients, the rest of the diet was similar between groups, except for grains. Although total grains intake was minimal at both baseline and at 4–6 months, we did find that the intervention group had a significantly higher intake compared to the control group. While whole grains may be a good source of vitamins and fiber starting at 6 months, the use of refined grains, especially before 6 months of age, may lead to excessive caloric consumption, increasing risk of rapid weight gain, and obesity (31). It is important to note that while the intervention messages encouraged parents to wait until 6 months to begin any cereal or solid foods, the use of cereal in milk bottles at a very young age was found to be common in this population. Previous studies have not assessed the effects of text message interventions on educating parents on complimentary foods and improving infant diet. The present intervention also included educational SMS messages on correct feeding practices, which were reported in a previous publication (32). Additionally, this population was also too young to appropriately measure the effect of some of the messages, such as reducing intake of sugar sweetened beverages.

It was expected that breastmilk intake would be higher in the intervention group as most messages were focused on breastfeeding. Also, as reported previously, frequency of breastfeeding did not increase with the intervention (32). Failure to see more significant differences in diet may be due to the nature of the intervention timing. For example, to influence breastmilk intake, the intervention may need to start before the decision to breastfeed is made but to detect differences in food groups and in other nutrients, the intervention may need to continue beyond 6 months, as other foods are introduced. Also, as published previously (32), many primary caregivers went back to work after 3 months, and infants were cared for by other family members that were not part of the intervention. One of the few studies using text messages, the LATCH study, which used two-way messaging to early communication, and the use of photo and video attachments among WIC participating mothers found small improvements in breastfeeding (33, 34). Similar methodology could be used to reinforce education on complimentary foods and improve infant diet quality.

The study included multiple strengths and limitations. Strengths of the study include the multi-site, longitudinal design conducted in WIC clinics with at-risk infants from underserved populations. The study also used a validated infant FFQ, which is uncommon in many studies assessing infant diet. While 24-h recalls are considered the gold standard for diet analysis, the validated FFQ has been shown to adequately represent infant diet. Additionally, the intervention was based on the TTM of behavior change which focused on and reinforced WIC feeding messages. Lastly, trained personnel were used to assess dietary intake. Limitations of the study include the short duration and not including infants past 6 months of age, when most foods are introduced. The study also did not include other caregivers that are directly involved in infant feeding and the intervention only included one SMS per week, more intense interventions may be needed to influence diet. We did not assess whether participants had previously participated in WIC programming, which may have affected the results. Future studies should start earlier (before birth) and continue until the infant is 1 year old and include more messages per week.

## Data availability statement

The data analyzed in this study is subject to the following licenses/restrictions: The datasets analyzed for this study are available upon request. Requests to access these datasets should be directed to CP, crpalaci@fiu.edu.

## Ethics statement

The studies involving human participants were reviewed and approved by University of Puerto Rico and University of Hawaii. Written informed consent to participate in this study was provided by the participants' legal guardian/next of kin.

## Author contributions

CP and JB designed the research. AM and SM conducted the research. AM analyzed the data. CP and AM wrote the paper. JB and MC were involved in interpreting the results and editing the manuscript. CP had primary responsibility for the final content. All authors contributed to the article and approved the submitted version.

## Funding

This study was supported by the National Institute of Minority Health and Health Disparities (NIMHD), of the National Institutes of Health under award number U54MD008149. Infrastructure support was also provided in part by the National Institute on Minority Health and Health Disparities RCMI Grant: 8G12MD007600. This research was also supported in part by grant U54MD007584 (RMATRIX) from the National Institute on Minority Health and Health Disparities (NIMHD) of the National Institutes of Health (NIH).

## Conflict of interest

The authors declare that the research was conducted in the absence of any commercial or financial relationships that could be construed as a potential conflict of interest. The reviewer AP declared a shared affiliation with the author MC at the time of review.

## Publisher's note

All claims expressed in this article are solely those of the authors and do not necessarily represent those of their affiliated organizations, or those of the publisher, the editors and the reviewers. Any product that may be evaluated in this article, or claim that may be made by its manufacturer, is not guaranteed or endorsed by the publisher.

## References

[1] Adair L. Long-term consequences of nutrition and growth in early childhood and possible preventive interventions. Nestle Nutr Inst Workshop Ser. (2014) 78:111–20. 10.1159/000354949 24504211

[2] Nader PR O'Brien M Houts R Bradley R Belsky J Crosnoe R. Identifying risk for obesity in early childhood. Pediatrics. (2006) 118:e594–601. 10.1542/peds.2005-2801 16950951

[3] Stettler N Zemel BS Kumanyika S Stallings VA. Infant weight gain and childhood overweight status in a multicenter, cohort study. Pediatrics. (2002) 109:194–9. 10.1542/peds.109.2.194 11826195

[4] Whitaker RC Wright JA Pepe MS Seidel KD Dietz WH. Predicting obesity in young adulthood from childhood and parental obesity. N Engl J Med. (1997) 337:869–73. 10.1056/NEJM199709253371301 9302300

[5] Young BE Johnson SL Krebs NF. Biological determinants linking infant weight gain and child obesity: current knowledge and future directions. Adv Nutr. (2012) 3:675–86. 10.3945/an.112.002238 22983846 PMC3648749

[6] Johnson B thorn B Mcgill B Suchman A Mendelson M Patlan KL. WIC Participant and Program Characteristics 2012. vol. AG-3198-C-. Alexandria, VA: U.S. Department of Agriculture, Food and Nutrition Service. (2013).

[7] (CDC) C for DC P. Behavioral Risk Factor Surveillance System Survey (BRFSS) data. (2009). Available online at: http://www.cdc.gov/BRFSS/.

[8] Torres R Santos E Orraca L Elias A Palacios C. Diet quality, social determinants, and weight status in Puerto Rican children aged 12 years. J Acad Nutr Diet. (2014) 114:1230–5. 10.1016/j.jand.2014.01.011 24656710 PMC4111983

[9] Soltero SM Palacios C. Association between dietary patterns and body composition in a group or Puerto Rican obese adults: a pilot study. P R Health Sci J. (2011) 30:22–7. 10.1016/j.nut.2010.02.011 21449494 PMC3449311

[10] Torres R Serrano M Perez CM Palacios C. Physical environment, diet quality, and body weight in a group of 12-year-old children from four public schools in Puerto Rico. P R Health Sci J. (2014) 33:14–21. 24665604 PMC4142497

[11] Lee SK Novotny R Daida YG Vijayadeva V Gittelsohn J. Dietary patterns of adolescent girls in Hawaii over a 2-year period. J Am Diet Assoc. (2007) 107:956–61. 10.1016/j.jada.2007.03.009 17524716

[12] Rios E Sinigaglia O Diaz B Campos M Palacios C. Development of a diet quality score for infants and toddlers and its association with weight. J Nutrit Health Food Sci. (2016) 4:1–7. 10.15226/jnhfs.2016.00171 28154840 PMC5283385

[13] Taveras EM Perkins M Woo Baidal JA Locks LM Cheng ER Blake-Lamb TL. The Impact of the First 1,000 Days on Childhood Obesity. Durham, NC. (2016). 26916260 10.1016/j.amepre.2015.11.010 PMC5207495

[14] Woo Baidal JA Locks LM Cheng ER Blake-Lamb TL Perkins ME Taveras EM. Risk factors for childhood obesity in the first 1,000 days: a systematic review. Am J Prev Med. (2016) 50:761–79. 10.1016/j.amepre.2015.11.012 26916261

[15] Johnson SL Gilley SP Krebs NF. Making every bite count: best practices for introducing foods during the complementary feeding period. Am J Clin Nutr. (2022). 10.1093/ajcn/nqac124 35678580 PMC9257526

[16] Irvine V John JR Scott JA Hayen A Do LG Bhole S. Factors influencing the early introduction of sugar sweetened beverages among infants: findings from the HSHK birth cohort study. Nutrients. (2020) 12:3343. 10.3390/nu12113343 33143073 PMC7693806

[17] Cartagena DC Ameringer SW Mcgrath J Jallo N Masho SW Myers BJ. Factors contributing to infant overfeeding with hispanic mothers. J Obstet Gynecol Neonatal Nurs. (2014) 43:139–59. 10.1111/1552-6909.12279 24502196

[18] USDA. DRI. Tables and Application Reports | Food and Nutrition Information Center | NAL | USDA. United States Departament of Agriculture. (2011).

[19] McGuire S. U.S. Department of Agriculture and U.S. Department of Health and Human Services. Dietary Guidelines for Americans, 2020-2025. Washington, DC: US Government Printing Office, January 2011. Adv Nutr. (2011) 2:293-4. 10.3945/an.111.000430 22332062 PMC3090168

[20] DeweyKGPannucciTRCasavaleKODavisTADonovanSMKleinmanRE. Development of food pattern recommendations for infants and toddlers 6–24 months of age to support the dietary guidelines for Americans, 2020–2025. J Nutr. (2021) 151:3113-24. 10.1093/jn/nxab20134195834

[21] Segura-Pérez S Calderón C Pérez-Escamilla R. Recomendaciones para la alimentación de la niña y el niño sano de 0 a 24 meses. Estrategias para prevenir el sobrepeso y la obesidad del infante y el niño pequeño San Juan, Puerto Rico. (2019).

[22] Ahluwalia N Herrick KA Rossen LM Rhodes D Kit B Moshfegh A. Usual nutrient intakes of US infants and toddlers generally meet or exceed dietary reference intakes: findings from NHANES 2009-2012. Am J Clin Nutr. (2016) 104:1167-74 10.3945/ajcn.116.137752 27629049 PMC6443261

[23] Poorman E Gazmararian J Parker RM Yang B Elon L. Use of text messaging for maternal and infant health: a systematic review of the literature. Matern Child Health J. (2014) 19:969–89. 10.1007/s10995-014-1595-8 25081242

[24] Séguin L Xu Q Potvin L Zunzunegui MV Frohlich KL. Effects of low income on infant health. CMAJ. (2003) 168:1533-8 12796331 PMC156683

[25] Banna J Campos M Gibby C Graulau RE Meléndez M Reyes A. Multi-site trial using short mobile messages (SMS) to improve infant weight in low-income minorities: Development, implementation, lessons learned and future applications. Contemp Clin Trials. (2017) 62:56–60. 10.1016/j.cct.2017.08.011 28827160 PMC5641256

[26] Prochaska JO Velicer WF. The transtheoretical model of health behavior change. Am J Health Promot. (1997) 12:38–48. 10.4278/0890-1171-12.1.38 10170434

[27] Food and Nutrition Services (FNS); US Department of Agriculture (USDA). Value Enhanced Nutrition Assessment (VENA) – WIC Nutrition Assessment Policy. WIC Policy Memo #2006-5 (2006).

[28] Palacios C Rivas-Tumanyan S Santiago-Rodríguez EJ Sinigaglia O Ríos EM Campos M. A semi-quantitative food frequency questionnaire validated in hispanic infants and toddlers aged 0 to 24 months. J Acad Nutr Diet. (2017) 117:526-35. 10.1016/j.jand.2016.12.010 28188114 PMC5382983

[29] Briefel RR Kalb LM Condon E Deming DM Clusen NA Fox MK. The Feeding Infants and Toddlers Study 2008: Study Design and Methods. J Am Diet Assoc. (2010) 110:S16-26 10.1016/j.jada.2010.09.005 21092765

[30] Nutrition Coordinating Center University University of Minnesota. Foods, Nutrients and Food Groups 2016. Available online at: http://www.ncc.umn.edu/about-ncc/foods-nutrients-and-food-groups/ (accessed April 30, 2016).

[31] Papanikolaou Y Fulgoni VL. Grain foods in US infants are associated with greater nutrient intakes, improved diet quality and increased consumption of recommended food groups. Nutrients. (2019) 11:2840. 10.3390/nu11122840 31756905 PMC6950092

[32] Palacios C Campos M Gibby C Meléndez M Lee JE Banna J. Effect of a multi-site trial using Short Message Service (SMS) on infant feeding practices and weight gain in low-income minorities. J Am Coll Nutr. (2018) 30:1–9. 10.1080/07315724.2018.1454353. 10.1080/07315724.2018.1454353 29708471 PMC6317355

[33] Harari N Rosenthal MS Bozzi V Goeschel L Jayewickreme T Onyebeke C. Feasibility and accepetability of a text message intervention used as an adjunct tool by WIC breastfeeding peer counsellors: the LATCH pilot. Mat Child Nutr. (2018) 14:e12488. 10.1111/mcn.12488 28766913 PMC6866153

[34] Martinez-Brockman JL Harari N Segura-Perez S Goeschel L Bozzi V Perez-Escamilla R. Impact of the Lactation Advice Through Texting Can Help (LATCH) Trial on Time to First Contact and Exclusive Breastfeeding among WIC Participants. J Nutr Ed Behav. (2018) 50:33-42. 10.1016/j.jneb.2017.09.001 29325660

